# Control of Multigene Expression Stoichiometry in Mammalian Cells Using Synthetic Promoters

**DOI:** 10.1021/acssynbio.0c00643

**Published:** 2021-05-03

**Authors:** Yash D. Patel, Adam J. Brown, Jie Zhu, Guglielmo Rosignoli, Suzanne J. Gibson, Diane Hatton, David C. James

**Affiliations:** † Department of Chemical and Biological Engineering, The University of Sheffield, Mappin Street, Sheffield, S1 3JD, U.K.; ‡ Cell Culture and Fermentation Sciences, BioPharmaceuticals Development, R&D, AstraZeneca, Gaithersburg, Maryland 20878, United States; § Dynamic Omics, Antibody Discovery & Protein Engineering, R&D, AstraZeneca, Cambridge, CB21 6GH, U.K.; ∥ Cell Culture and Fermentation Sciences, BioPharmaceuticals Development, R&D, AstraZeneca, Cambridge, CB21 6GH, U.K.

**Keywords:** gene expression, synthetic promoter, transcriptional interference

## Abstract

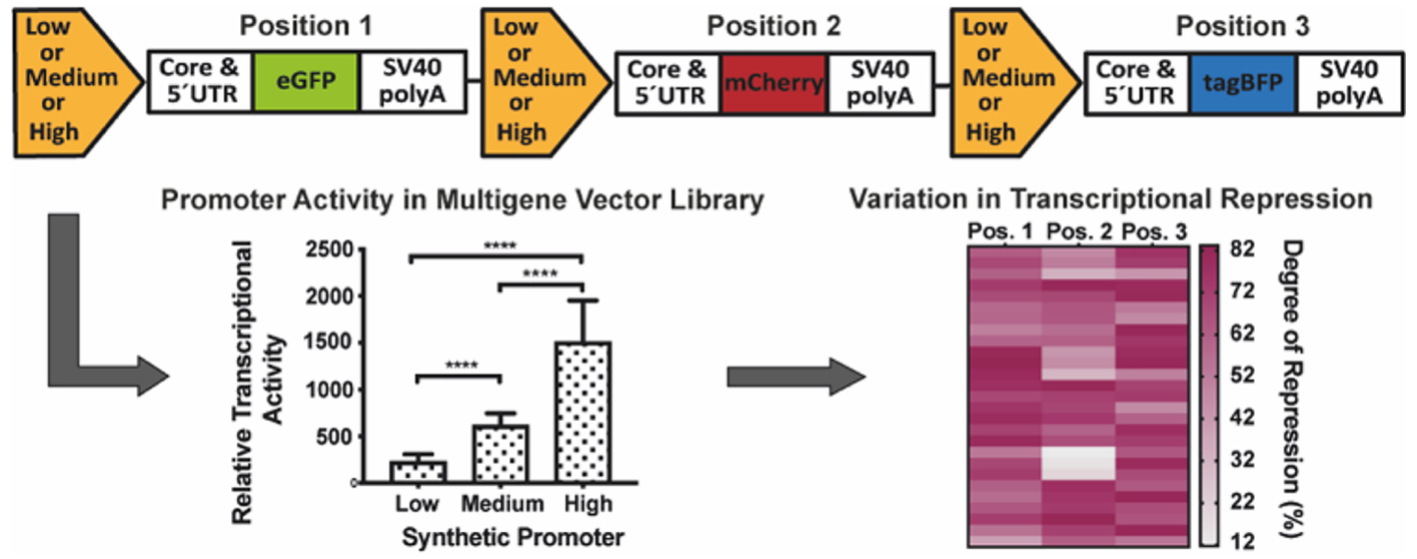

To successfully engineer mammalian cells for a desired purpose, multiple recombinant genes are required to be coexpressed at a specific and optimal ratio. In this study, we hypothesized that synthetic promoters varying in transcriptional activity could be used to create single multigene expression vectors coexpressing recombinant genes at a predictable relative stoichiometry. A library of 27 multigene constructs was created comprising three discrete fluorescent reporter gene transcriptional units in fixed series, each under the control of either a relatively low, medium, or high transcriptional strength synthetic promoter in every possible combination. Expression of each reporter gene was determined by absolute quantitation qRT-PCR in CHO cells. The synthetic promoters did generally function as designed within a multigene vector context; however, significant divergences from predicted promoter-mediated transcriptional activity were observed. First, expression of all three genes within a multigene vector was repressed at varying levels relative to coexpression of identical reporter genes on separate single gene vectors at equivalent gene copies. Second, gene positional effects were evident across all constructs where expression of the reporter genes in positions 2 and 3 was generally reduced relative to position 1. Finally, after accounting for general repression, synthetic promoter transcriptional activity within a local multigene vector format deviated from that expected. Taken together, our data reveal that mammalian synthetic promoters can be employed in vectors to mediate expression of multiple genes at predictable relative stoichiometries. However, empirical validation of functional performance is a necessary prerequisite, as vector and promoter design features can significantly impact performance.

Mammalian cells utilize complex, finely tuned gene networks to maintain essential cellular functions.^1−3^ To genetically engineer these networks for biomedical and therapeutic applications,^2,4−6^ it will ultimately be necessary to precisely control coexpression of multiple recombinant genes simultaneously. While single plasmids encoding multiple transcriptional units (TUs) in series can be constructed using Gibson or Golden Gate assembly technology with relative ease,^7−12^ control of the relative level at which several individual genes are constitutively expressed to achieve a desired stoichiometry is far more difficult. Current methods to achieve controlled expression of recombinant genes in mammalian cells employ multiple single gene synthetic circuits cooperatively functioning using inducible systems and complex gene switches.^13−15^ However, constitutively controlling expression of multiple genes simultaneously at different stoichiometries on a single plasmid could be a simpler approach for *in vivo* systems and engineering.

Recombinant gene expression within synthetic circuits can be precisely controlled using an assortment of oscillatory, logic gates^13^ and feedback loops.^16^ However, this frequently involves the application of synthetic transcription factors, such as transcription activator-like effectors (TALEs),^17^ zinc fingers,^18^ chimeric transcription factors,^19^ or CRISPR transcription factors^20^ to induce cognate promoters. Alternatively, chemical chaperones,^21^ aptamers,^22^ metabolites,^19^ and other external stimuli^15^ have all also been employed to induce and regulate synthetic gene circuit expression. These sophisticated biological control systems can be useful, but are also complex and unwieldy, with expression levels determined by ligand (synthetic transcription factors and chemical chaperones) concentration dependent transactivation or repression and the potential of imprecise and leaky expression.^16,23^ While complex, programmable gene expression systems will be required for many applications, “hardwired” components operating at constitutive fixed stoichiometries generally form the basis of all engineered systems.

An alternative means to control recombinant gene expression stoichiometry is the use of synthetic promoters with defined transcriptional activity.^24^ In this case a promoter can be specifically designed to utilize the host cell’s existing repertoire of transactivators to a varying extent in order to achieve a desired level of transcriptional activity.^24−26^ As a means to control multigene expression stoichiometry, the use of well-defined synthetic promoters in vector constructs is therefore a potentially attractive solution.

In this study, we tested the hypothesis that constitutive synthetic promoters varying in transcriptional activity could be used to create discrete multigene expression vectors (MGEVs) coexpressing recombinant genes at a predictable relative stoichiometry in CHO cells. We reveal that mammalian synthetic promoters varying in transcriptional strength can be used to achieve variable multigene expression stoichiometry. However, multigene coexpression is inherently context-specific and promoter and vector design features can significantly affect predicted performance.

## Results and Discussion

### Transient Coexpression of Reporter Genes Using Synthetic Promoters in a Single Gene Per Plasmid Vector Format

**Figure 1:**
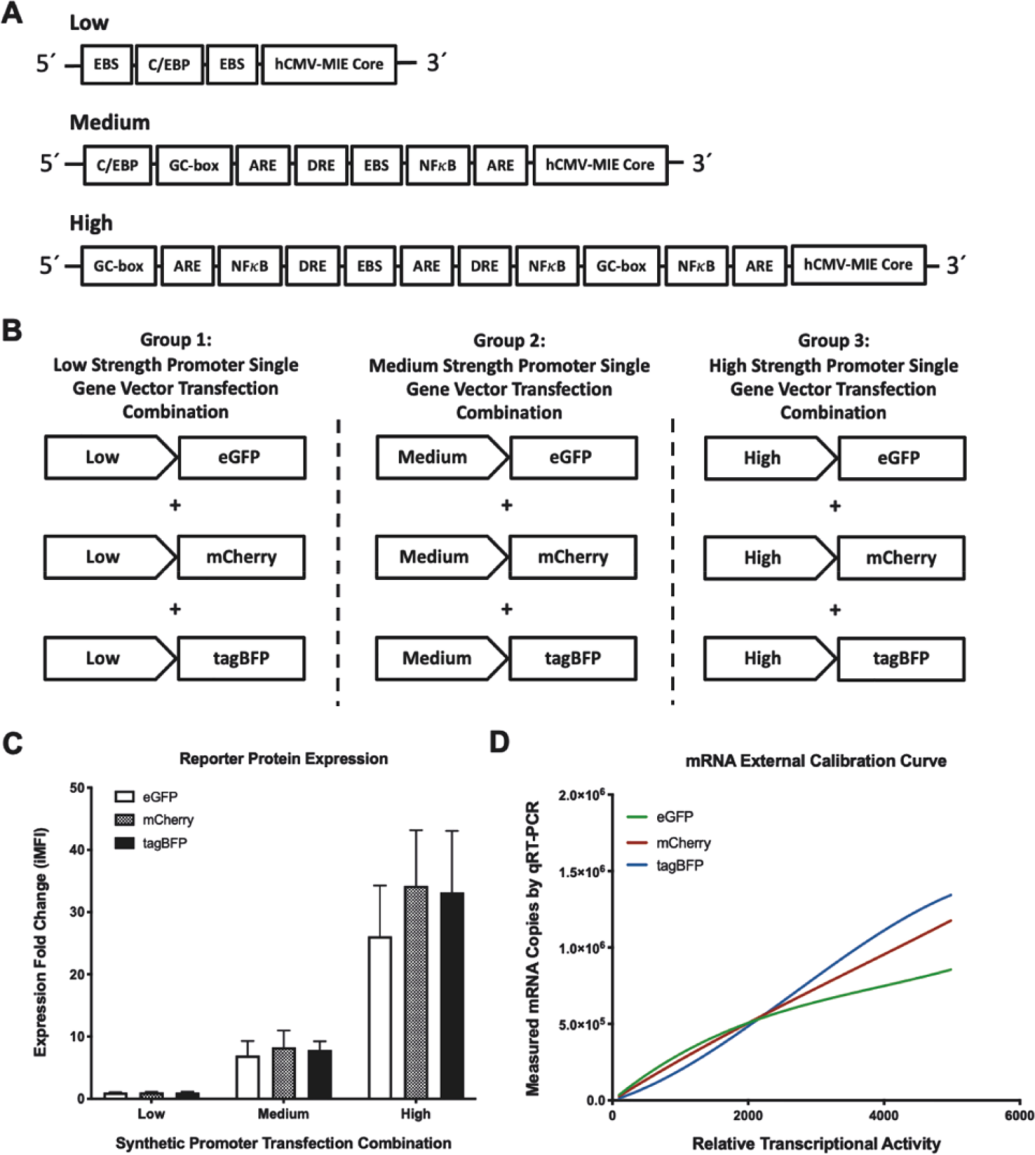
Coexpression of three fluorescent reporter proteins to evaluate synthetic promoter activity. (A) The transcription factor regulatory element (TFRE) composition of the mammalian synthetic promoters utilized in this study. The TFRE blocks separated by a 2 bp spacer were specifically selected for the low, medium, and high strength promoters and positioned upstream of the human cytomegalovirus major intermediate-early (hCMV-MIE) core in order to vary each promoter’s transcriptional activity. The low, medium, and high strength synthetic promoter’s approximate activity was 0.1, 0.8, and 2.2-fold of hCMV-MIE expression strength, respectively.^24^ The list of TFRE abbreviations are as follows: antioxidant regulatory element (ARE), CCAAT-enhancer binding protein (C/EBP), dioxin regulatory element (DRE), ETS binding site (EBS), nuclear factor kappa B (NFκB). (B) The low, medium, and high strength synthetic promoters were cloned upstream of three spectrally distinct fluorescent reporters (eGFP, mCherry, and tagBFP) creating a library of nine single gene vectors (SGVs). These were divided into three transfection groups according to their respective strengths: Group 1, low strength; Group 2, medium strength; and Group 3, high strength. Each group comprised three cotransfected SGVs at equimolar quantities ranging from 100 to 800 ng of total DNA load, in order to evaluate the synthetic promoter’s activity. (C) The mammalian synthetic promoter activity determined by relative fluorescent reporter expression fold change. The fold change was derived by normalizing the integrated median fluorescent intensity (iMFI) detected for each reporter utilizing the medium and high strength promoters relative to the low strength promoter. An expression fold change was derived for each total DNA load (100 to 800 ng) transfected, and the average fold change for eGFP, mCherry, and tagBFP are represented by the white, gray, and black bars, respectively. The error bars indicate the standard deviation of reporter expression fold change across all the total DNA loads transfected over three independent experiments. (D) The external calibration curve was constructed to normalize different fluorescent reporter mRNA copies for direct comparison within a multigene expression vector (MGEV) context. The calibration curve was derived by arithmetically combining mRNA copies detected at each DNA load (100 to 800 ng) and different promoter strengths while normalizing to the low strength data set. A third-order polynomial regression curve was fitted to model the different mRNA dynamics of eGFP (green), mCherry (red), and tagBFP (blue), and yield a normalized relative transcriptional activity (RTA) for each promoter–reporter combination. The *r*^2^ of the third-order polynomial curves for eGFP, mCherry, and tagBFP were 0.976, 0.988, and 0.964, respectively.

Three heterotypic mammalian synthetic promoters previously designed to provide a relatively low, medium, and high level of gene expression were selected from a library developed by Brown et al.^24^ Synthetic promoter transcription factor regulatory element (TFRE) composition is shown in Figure 1A. The promoters comprised a selection of up to six different TFREs varying in transcriptional activity as previously characterized in CHO cells.^24^

To confirm synthetic promoter transcriptional activity and quantitatively evaluate recombinant gene expression from single gene vectors (SGVs) for subsequent comparison with gene expression from MGEVs, each synthetic promoter was inserted upstream of three spectrally discrete fluorescent reporter proteins, eGFP, mCherry, and tagBFP to create a library of nine SGVs (Supporting Information, Figure S1). These were cotransfected in three groups, each group consisting of three SGVs encoding each fluorescent protein under the control of the same transcriptionally active promoter, either low (group 1), medium (group 2), or high (group 3), at a total plasmid DNA load ranging from 100 to 800 ng (Figure 1B). As expected, SGV-mediated transient expression of each reporter gene resulted in a relatively low, medium, or high cellular content of fluorescent protein dependent upon the synthetic promoter utilized. Across all reporters, normalized (relative to low strength promoter data) median fluorescence intensities were in the ratio 1:7.7:31.2 (low/medium/high strength synthetic promoters), confirming expected promoter functionality (Figure 1C).

However, as the sensitivity of flow cytometric detection of fluorescent proteins at low expression levels was limited, and in order to quantitatively compare transcriptional activity more directly, we measured recombinant cellular reporter mRNA content using absolute quantitation qRT-PCR. To externally calibrate measured mRNA copies to variation in transcriptional activity for each reporter, recombinant mRNA copies derived from a range of transfected SGV total plasmid DNA loads (over the range 100 to 800 ng per 1.86 × 10^6^ cells) were measured by qRT-PCR. For each experiment an equal mass of eGFP, mCherry, and tagBFP SGVs utilizing either low, medium, or high strength promoters were mixed prior to transfection such that all reporters were cotransfected as either low, medium of high strength synthetic promoter groups as previously described (Figure 1B). As each reporter gene was a similar length (eGFP, 720 bp; mCherry, 711 bp; tagBFP, 702 bp) the number of copies of each reporter gene cotransfected in each experiment was similar, for example, 600 ng total plasmid DNA equals 26983–27868 copies of each fluorescent reporter gene per cell. At each total SGV DNA load, reporter mRNA copies were measured 24 h after transfection by qRT-PCR. A linear relationship was observed between average reporter mRNA copies and total SGV DNA load (100 to 800 ng) for each transfection group (Figure 1B) indicating detection by qRT-PCR was within the dynamic range for each promoter–reporter combination (Figure S2). To enable direct comparison of transcriptional activities for each reporter mRNA measurement, mRNA copies measured at each DNA load and varying promoter strength were arithmetically combined and normalized with respect to the low strength promoter data sets, incorporating the assumption that while reporter-specific mRNA dynamics likely vary post-transcription (i.e., mRNA half-life, mRNA secondary structure, translation efficiency, etc.)^27−31^ the transcriptional rate mediated by a given promoter was constant for each reporter gene. This enabled reporter-specific external calibration curves to be created, relating measured mRNA copies to a cross-reporter comparable relative transcriptional activity (RTA). In each case, a third-order polynomial regression provided the line of best fit (*r*^2^ of 0.976, 0.988, and 0.964 for eGFP, mCherry, and tagBFP calibrations, respectively; Figure 1D). Unsurprisingly, reporter-specific differences in measured mRNA copies and RTAs were apparent, indicative of differences in mRNA dynamics despite the use of common 5′ (AZ’s proprietary 5′ untranslated region (UTR)) and 3′ (simian virus 40 (SV40) late polyadenylation (polyA) element) UTRs in each case. Across all SGV data (accounting for every total DNA load), synthetic promoters yielded RTAs in the normalized mean (±SE) ratio low 1:medium 4.6 (±1.1):high 7.2 (±1.1). The ratio of synthetic promoter activities was to an extent dependent upon total plasmid DNA load, such that relative to the low strength promoter, the medium and high ratios increased linearly with mass of transfected DNA (Figure S3), potentially indicative of reduced self-inhibition (also referred to as promoter interference) with increased promoter complexity at higher DNA loads.

### Construction and Performance of Multigene Expression Vectors Utilizing Synthetic Promoters to Control Recombinant Gene Expression Stoichiometry

**Figure 2:**
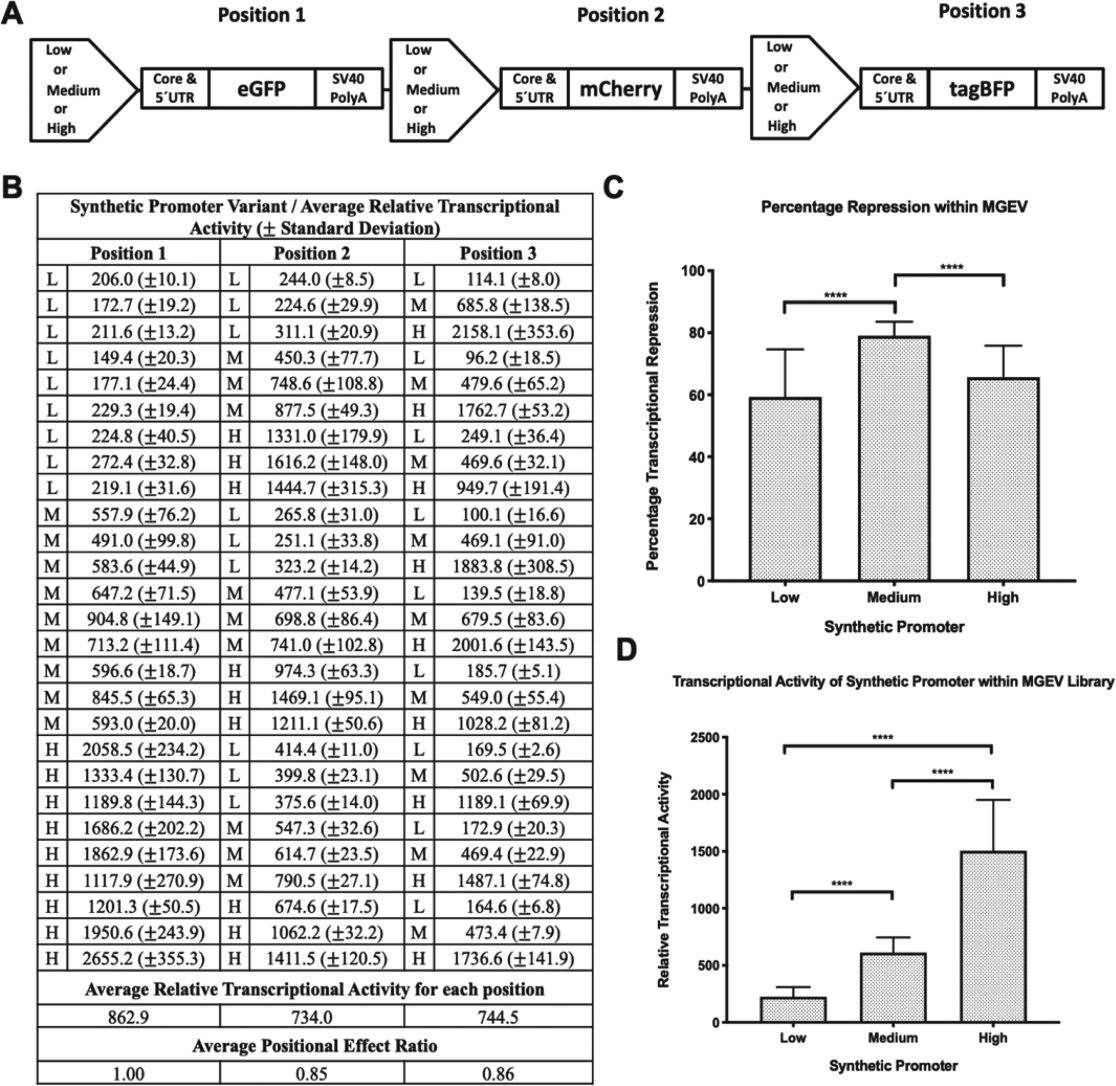
Multigene expression vectors (MGEVs) utilizing mammalian synthetic promoters to control recombinant gene expression stoichiometry. (A) A library of 27 MGEV variants was constructed encoding eGFP, mCherry, and tagBFP in a fixed tandem series utilizing a low, medium, and high strength mammalian synthetic promoter in each position encompassing every possible combination. The core promoter and untranslated regions (UTRs) were identical in each transcription unit (TU) within the MGEV-hCMV-MIE core, AstraZeneca’s proprietary 5′UTR and SV40 late polyA. All MGEVs were constructed by Golden Gate assembly using *de novo* synthesized TUs and a pExp-Vec-GG backbone (refer to Figure S1). (B) The normalized reporter mRNA copies detected for each MGEV variant after 24 h expression and depicted as average relative transcriptional activity (RTA) ± standard deviation of three independent experiments. The RTA was derived by interpolating against the single gene vector (SGV) external calibration curves (Figure 1D) to allow direct comparison of reporter expression. The low, medium, and high strength synthetic promoters utilized in each position of the MGEV variants were abbreviated to “L”, “M” and “H”, respectively. A mean RTA for position 1, 2, and 3 across the 27 MGEV variants was calculated, and an overall gene positional effect ratio was derived by normalizing the mean RTA in position 2 and 3 relative to position 1. (C) The average transcriptional repression of the low, medium, and high strength synthetic promoter exhibited during transient expression of the MGEV library. The percentage transcriptional repression for each synthetic promoter was calculated by comparing the difference between the RTAs observed during MGEV expression and expected RTAs derived from SGV coexpression (Figure 1B) at roughly equivalent gene copies. The individual bars and error bars represent the average percentage transcriptional repression and standard deviation respectively for the low, medium, and high strength promoter across all positions within the MGEV (27 discrete RTAs per promoter variant) across three independent experiments. A one-way ANOVA statistical test with a Tukey correction was performed to show a significant difference in average percentage transcriptional repression between the low and medium, and medium and high strength promoters and represented by “****” for *p* < 0.0001. (D) The average RTA of the low, medium, and high strength synthetic promoter utilized across the 27 discrete MGEV variants irrespective of position. The error bars represent a standard deviation of 27 individual RTAs for each promoter across three independent experiments. A one-way ANOVA statistical analysis with a Tukey correction was performed to show significant differences between the low, medium, and high strength synthetic promoters and represented by “****” for *p* < 0.0001.

We tested the hypothesis that synthetic promoters could be used to predictably control the relative level of expression of recombinant genes arranged in series in MGEVs. We constructed a library of 27 MGEVs encoding eGFP, mCherry, and tagBFP in a fixed series utilizing all possible combinations of synthetic promoters (low, medium, and high) in the different positions within the series, while keeping the core (hCMV-MIE core), and the 5′ (AZ’s proprietary UTR) and 3′ (SV40 late polyA) UTRs constant, as shown in Figure 2A. Each MGEV was constructed by Golden Gate assembly^32−34^ using the *de novo* synthesized TUs and plasmid vector backbone pExp-Vec-GG (Figure S1).

Each MGEV variant was transfected into CHO cells for 24 h as per the SGV combinations (Figure 1B) at a total MGEV mass of 600 ng per 1.86 × 10^6^ cells. Under these conditions, the number of fluorescent gene copies transfected (29113 ± 212 copies of each fluorescent reporter gene per cell) was approximately equivalent to the number of gene copies transfected using 600 ng of combined SGV vector plasmid DNA (27 489 copies of each reporter per cell, see above) and within the linear range (Figure S2). Therefore, derived from SGV expression data at the same plasmid DNA load employed, the predicted RTAs for low, medium, and high strength synthetic promoters, respectively, were 552 (±20), 2915 (±284), and 4384 (±874), respectively, a ratio of 1:5.3:7.9. For each MGEV, reporter mRNA copies were measured by qRT-PCR and RTAs derived using the SGV external calibration (Figure 1D). These data are listed in Figure 2B.

The most obvious general trend observed was a substantial overall repression of reporter gene transcription relative to that observed using SGVs (overall mean of 69.9% relative to SGV mediated transcription). This repression is quantified per synthetic promoter in Figure 2C and a comparison of relative promoter-mediated transcriptional activity is shown in Figure 2D (in both cases across all combinations utilized). These data clearly reveal that in a MGEV context synthetic promoters did generally yield the expected transcriptional trend (i.e., L < M < H; Figure 2D), although the actual ratio (1:2.8:6.7) was different from that obtained using SGVs (1:5.3:7.9). Together, the data show more marked repression of the medium strength synthetic promoter (Figure 2C). We infer that overall transcriptional repression may be attributed to change in plasmid structure by negative and positive supercoiling, where the plasmid conformation pre- and post-RNA polymerase II (RNA pol II) transcription elongation can hinder localized gene transcription.^35−37^ Additionally, the potential bidirectional behavior of promoters^38,39^ in a fixed tandem series can lead to antisense transcription and RNA pol II collisions, in turn inhibiting the transcription of neighboring TUs.^36,40^ Both these mechanisms could be concurrently contributing toward the general repression within a MGEV context. Moreover, the bacterial sequences (β-lactamase gene and origin of replication) within the vector backbone of both the MGEVs and SGVs were identical, hence potential bacterial sequence related transgenes silencing^41^ causing differences in transcriptional repression in a MGEV context compared to SGVs is unlikely. Furthermore, other studies have reported that transgene silencing may not be bacterial sequence specific.^41^

Gene positional effect within the library of vectors was quantified simply by summation of all RTAs deriving from positions 1, 2, and 3 (i.e., using all synthetic promoter combinations; Figure 2B). This revealed that maximum reporter expression occurred at position 1. Relative to position 1, positions 2 and 3 exhibited a 15% and 14% reduction in reporter gene transcription, respectively. We hypothesize that the gene positional effect is a consequence of inefficient transcription termination of the upstream TU causing transcriptional read through of the RNA pol II elongation complex into the neighboring TU. This limits binding of transcription factors or assembly of the preinitiation complex by steric hindrance in the promoter region of the TU inhibiting transcription initiation. The mechanism is referred to as occlusion-mediated transcriptional interference.^40,42^ Alternatively, the RNA pol II elongation complex can also dislodge transcription factors bound to the enhancer region of a downstream promoter resulting in repressed transcription.^42^ Other dual promoter systems in tandem arrangement within a standard or lentiviral vector have also exhibited unpredictable gene expression both caused by transcriptional interference.^43,44^ Similarly, a triple gene cassette constructing a synthetic pathway in *Saccharomyces cerevisiae* also exhibited substantial discrepancies from predicted expression attributed to transcriptional interference.^45^

### Bias in Recombinant Gene Transcription in a Multigene Vector Context

We further analyzed the observed RTA for each of the 27 MGEV variants (Figure 2B) to discern specific biases in recombinant gene transcription within a multigene context. This was achieved by comparing the “observed RTA” within a MGEV against a set of “expected RTAs” derived from SGV coexpression at approximately equivalent gene copies.

**Figure 3:**
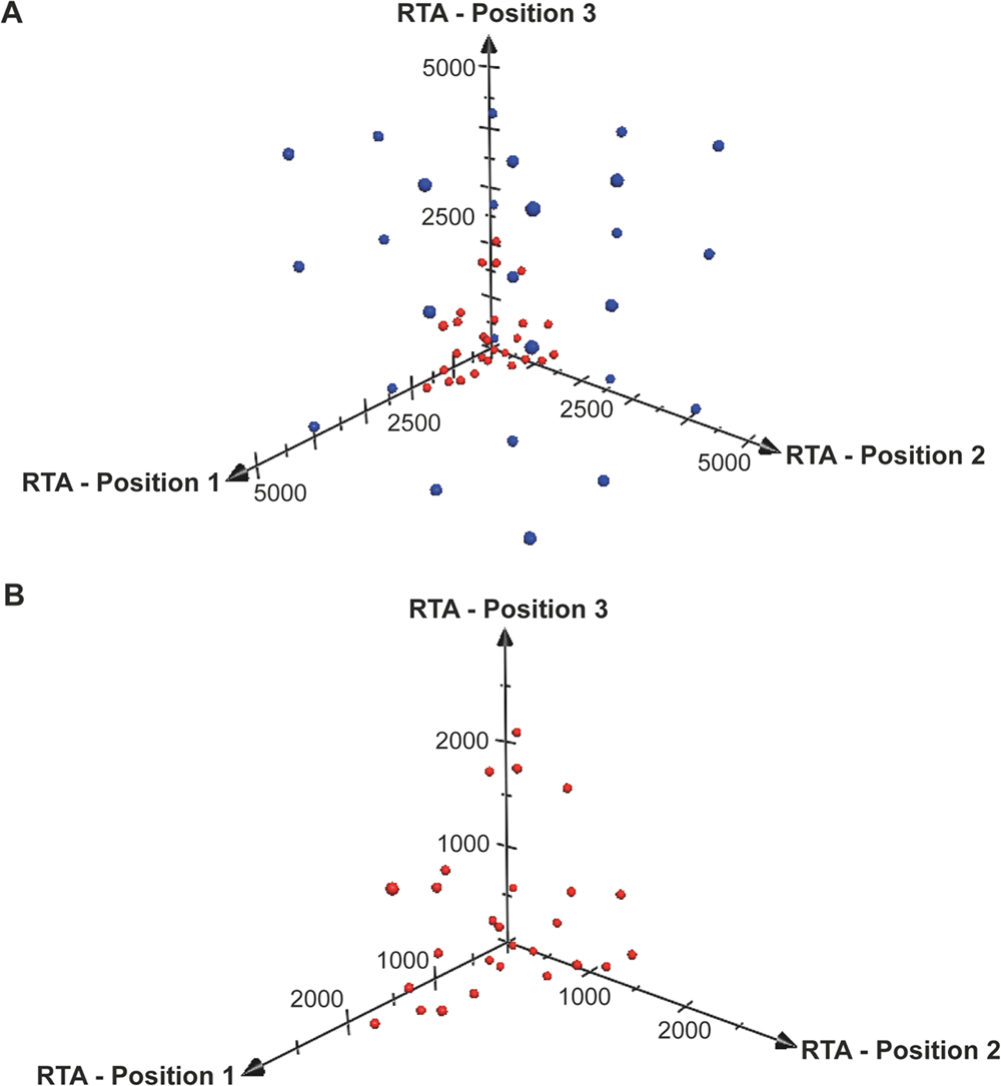
Overview of transcriptional activity within a multigene expression vector (MGEV) context. (A) The three-dimensional plot depicts the relative transcriptional activity (RTA) in positions 1, 2, and 3 across the *x*, *y*, and *z* axis respectively for 27 discrete MGEV variants utilizing a low, medium, and high strength synthetic promoter in every combination and position within the MGEV and represented as red points. A set of RTAs for a low, medium, and high strength synthetic promoter was derived from single gene vector (SGV) coexpression at approximately equivalent gene copies (Figure 1B) and compiled to simulate the expected transcriptional activity in each position of a MGEV. These RTAs were represented as blue points on the plot. (B) A magnified plot representing the same RTAs in position 1, 2, and 3 of the 27 MGEV variants as shown in panel A. The cluster indicates the empirically derived limits of transcriptional activity of the low, medium, and high strength synthetic promoters within the context of a MGEV.

We compared the observed RTA of each position within a MGEV to its expected RTA counterpart within a three-dimensional plot as shown in Figure 3, where each axis represented one of the three positions within the MGEV. Figure 3A reiterates the substantial transcriptional repression in all positions for each MGEV as shown by the clustered conformation of the observed RTAs (ranging from 96.2 to 2655.2) compared to the cubic conformation of the expected RTA (ranging from 551.9 to 4383.5). The cluster of MGEV RTAs (Figure 3B) was asymmetrical with lower overall transcriptional activity observed in position 2 across 27 discrete variants (mean RTA of 734.0) re-emphasizing increased general transcriptional repression compared to positions 1 and 3. We rationalize that Figure 3B depicts the empirically derived design space for achievable transcriptional activity of three recombinant genes in a fixed tandem series utilizing a low, medium, and high strength synthetic promoter accounting for a range of potential transcriptional interfering mechanisms.

**Figure 4:**
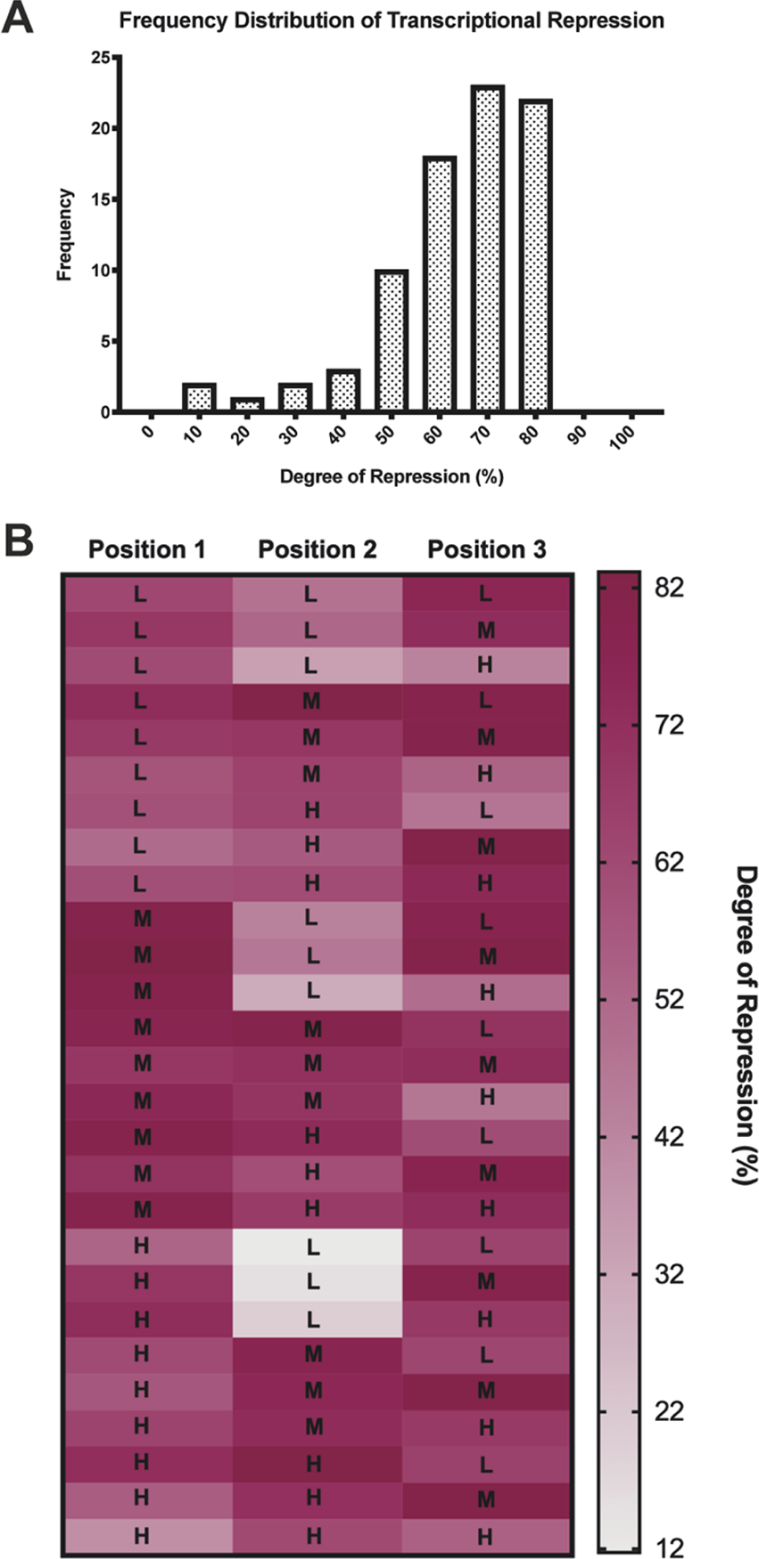
Trends in transcriptional repression within a multigene expression vector (MGEV) context. (A) A frequency distribution representing the degree of transcriptional repression across all three positions within the library of 27 MGEV variants. The relative transcriptional activity (RTA) detected within a MGEV was normalized for overall gene positional effects by compensating for the 15% and 14% repression observed in positions 2 and 3, respectively (Figure 2B) so to identify other contributing biases in transcriptional activity. These positional effect normalized RTAs for each position and every synthetic promoter combination (low, medium, and high strength) were directly compared against expected RTAs (derived from single gene vector coexpression at approximately equivalent gene copies (Figure 1B)) to yield a percentage in transcriptional repression. The degree of repression was then categorized into fixed intervals ranging from 0 to 100% as individual bin centers and the frequency calculated for each interval to form a distribution. (B) The degree of repression calculated in panel A was arranged according to the synthetic promoter combination and position within the 27 discrete MGEV variants. A color gradient heat map was constructed to represent the degree of transcriptional repression, and it indicates specific trends in synthetic promoter transcriptional activity. Shades of purple represent high repression, conversely shades of gray represent lower repression. The synthetic promoter utilized in the specific position is overlaid and abbreviated as “L”, “M” and “H” representing low, medium and high strength, respectively.

To identify other transcriptional repression trends within the MGEV library, we normalized for the overall observed gene positional effect (15% and 14% repression in positions 2 and 3, respectively) for the detected RTA in position 2 and 3 of each MGEV. These positional effect normalized RTAs from MGEV expression were compared against expected RTAs (derived from SGV coexpression at roughly equivalent gene copies) yielding a percentage of transcriptional repression for each position. The distribution of transcriptional repression irrespective of position showed that the majority (90.1%) of expression was repressed by >50% and the median transcriptional repression was 68.6% (Figure 4A).

Positional or promoter specific gene repression trends were highlighted in Figure 4B. The color gradient heat map depicts the degree of repression relative to the expected RTAs for each position across the MGEV library. The medium strength synthetic promoter consistently demonstrated repressed activity with an average transcriptional repression of 76.5% (12% higher than the mean transcriptional repression observed). Conversely, the low strength synthetic promoter exhibited enhanced transcriptional activity when neighboring a higher strength synthetic promoter where the degree of repression (48.2%) was lower than the average (64.5%). The promoter activity was particularly higher in position 2 with an average transcription repression of 31.7%. Generally, the high strength promoter did not exhibit any specific transcriptional trends but broad context-specific variation was evident for which repression ranged from 39.4 to 81.9%.

We rationalize that the deviation of promoter activity (after accounting for general repression) is context-specific to the localized environment within a MGEV where promoter squelching may be impacting transcription. Promoter squelching refers to competition of transcription factors and associated cofactors involved in regulating transcription between promoter variants resulting in bias gene expression activity.^46−48^ When referring to the TFRE composition of the low, medium, and high strength synthetic promoter, all six transcription factors and their cognate TFREs are shared between the promoter variants (Figure 1A). The medium strength synthetic promoter shares TFRE-blocks with both the low (EBS and C/EBP) and high (GC-box, ARE, DRE, EBS, NFκB) strength synthetic promoter, which may indicate increased competition for transcription factors. However, in general as reporter gene expression was within a linear range (Figure S2) with respect to gene dosage, it is unlikely that competition for transcription factors was caused by saturation of transcriptional activity from recombinant genes. This would suggest the repressed state of the medium strength promoter is potentially caused by squelching. The enhanced activity of the low strength synthetic promoter neighboring a higher strength synthetic promoter variant could be caused by interaction between transcription factors. Literature has shown cofunctionality between derivatives of the C/EBP and NFκB transcription factors to initiate transcription in immune and cancer cells.^49,50^ We speculate that the abundance of NFκB and its potency on transcription rate,^24,51^ and interaction with C/EBP transcription factors, may be enhancing transcription of the low strength synthetic promoter within the local MGEV environment.

## Conclusion

Our data can be used to aid *de novo* design of MGEVs for future applications. The major factor impacting nonpredictable recombinant gene expression performance was general repression of colocated transcriptional units utilizing synthetic promoters. A solution to this could derive from a consideration of two related design criteria.

First, synthetic promoter design could be deliberately more complex, effectively increasing the diversity of TFREs and thus the available transcription factor repertoire that can drive expression of recombinant genes, thus minimizing promoter–promoter competitive interference. More complex, larger viral promoters such as CMV exhibit lower self-repression within a MGEV context,^51,52^ where broad access to the host cell’s transcriptional landscape is clearly a functional advantage *in vivo*. A variation of this design criterion would be the use of synthetic promoters composed of different TFREs to avoid competitive interference.

Second, spatial organization and isolation of transcriptional units should be improved to minimize positional and repressive effects. Co-located assemblies of synthetic DNA elements (an underpinning concept) may be inherently susceptible to corepression through steric limitations on transcription initiation.^40,42^ While other solutions that employ different means to simultaneously deliver multiple transcription units could be utilized (e.g., artificial chromosomes,^53^ transposases^54,55^), in the current context (delivery of plasmid DNA), a simpler practical solution could be the inclusion of efficient transcription terminator and insulator elements within the MGEV^44,56^ to avoid, for example, transcriptional run-through.^57^ For example, a β-globin cotranscriptional cleavage (CoTC) terminator element has been shown to improve transcription termination efficiency and potentially reduce transcriptional interference,^58^ whereas a chicken hypersensitivity site 4 (cHS4) insulator would avoid distal promoter-mediated transcription by functioning as a enhancer-blocker.^59−61^ However, both of these elements are large (ranging from 800 to 1200 bp)^62,63^ resulting in increased plasmid size—risking cellular toxicity and poor transfectability.^64,65^ More recently, as part of their development, shorter insulators which include the core CCCTC-enriched elements (250 bp) derived from the cHS4 element have maintained insulator functionality and could be a viable option to alleviate positional effects.^59,62^ We believe future studies exploring different transcription unit orientations such as a divergent conformation and inclusion of efficient transcription terminators and insulators within a MGEV context would reveal transcriptional interference mechanisms contributing to unpredictable expression. Moreover, we emphasize that while we show that synthetic promoter technology can be used to control relative expression of individual recombinant genes encoded in a MGEV, additional testing of MGEV performance in a particular context is essential (i.e., stable expression, host cell type), and specific to the application.

The most advanced natural MGEV systems are arguably viruses—natural biological systems specifically designed for compact encoding of complex multimeric assemblies and functions.^66,67^ For example, adeno-assisted viruses (AAVs) achieve a precise expression stoichiometry of multiple genes (7) within a compact genome (∼5 kb).^67,68^ This is achieved using a combination of internal (within open-reading frame) promoters, differential mRNA splicing, overlapping open reading frames (ORFs), varying translation initiation rates (by varying start codons) and feedback loops (using transactivators or repressors).^67−69^ Combined aspects of these control systems could be employed in synthetic DNA assemblies to achieve predictable coexpression performance; however, this would be entirely dependent on the desired application. Most synthetic systems would require coexpression of multiple distinct open reading frames encoding different, nonoverlapping functions.

In conclusion, our study highlights the importance of genetic engineering platform design systems. While it is entirely possible to rapidly synthesize and assemble discrete DNA elements, whole synthetic assembly functionality requires platform engineering control systems and parts that enable essential system performance parameters such as relative expression level and stoichiometry to be predictably embedded.

## Materials and Methods

### Single and Multigene Expression Vector Library Construction

The transcription units (TUs) were designed in-house and *de novo* synthesized by GeneArt (Regensburg, Germany) comprising a human cytomegalovirus major-intermediate early (hCMV-MIE) core promoter, a fluorescent protein coding DNA sequence (CDS) for eGFP, mCherry and tagBFP, optimized for expression in *Cricetulus griseus* (Chinese hamster), and a simian virus 40 (SV40) late polyadenylation sequence (Figure S1). The proximal region of the synthetic promoters developed by Brown et al.^24^ exhibiting a relatively low, medium, and high level of transcriptional activity within CHO cells were PCR modified to facilitate subcloning into the TUs by restriction digestion–ligation cloning upstream of the hCMV-MIE core element. The MGEV library was assembled by cloning the TUs into a recipient expression vector called pExp-Vec-GG (which was *de novo* synthesized by GeneArt comprising a glutamine synthetase (GS) expression cassette regulated by an SV40 promoter, a mammalian episomal origin of replication element, and a β-lactamase gene for ampicillin resistance selection; Figure S1). This was performed by using the Golden Gate assembly kit (New England Biolabs, Hitchin, UK) according to the manufacturer’s protocol. Successful SGV and MGEV constructions were identified by a restriction digest of purified DNA.

### High Throughput 96-Well Transient Transfection and Culturing

Transient expression was performed in a CHO-K1 host cell line adapted to growth in suspension in chemically defined medium, hereby referred to as CHO cells. Three biological replicates were performed in this study where each biological replicate comprised three technical replicates. In each case, the cells were cultured prior to transfection in CD CHO medium (Thermo Fisher Scientific, Paisley, UK) supplemented with 6 mM l-glutamine (Thermo Fisher Scientific), hereby described as CD CHO culture medium, for 72 h. Transfection was performed by electroporation using the Amaxa Nucleofector system (Lonza, Basel, Switzerland) coupled with the Nucleofector 96-well Shuttle system (Lonza) for high throughput (HT) transfection according to manufacturer’s protocol. Each reaction comprised either 600 or 800 ng DNA (either a combination of SGVs encoding for eGFP, mCherry, and tagBFP and noncoding DNA, or a MGEV plasmid encoding for the same three fluorescent proteins) resuspended into 2.5 μL of nuclease-free d.H_2_O (Qiagen) combined with 7.5 μL of nucleofection solution (prepared according to Amaxa SG Cell Line IV 96-well electroporation kit instructions (Lonza)). A total of 1.86 × 10^6^ cells were prepared by resuspending into 10 μL of nucleofection solution. The DNA and cells were combined and 20 μL of the DNA-cell mix was transferred into a 96-well Nucleocuvette plate (Lonza) and electroporated using the program FF-158. The transfected cells per well were recovered by the addition of 80 μL of prewarmed CD CHO culture medium. The transfected cells were cultured by seeding 20 μL of transfectants into 180 μL of prewarmed CD CHO culture medium per well (10-fold dilution) into a 96-well culture plate (Thermo Fisher Scientific). The culture plate was incubated at 37 °C, 5% (v/v) CO_2_ with humidity for 24 h prior to quantification by qRT-PCR or flow cytometry.

### Harvesting Transfected Cells, RNA Extraction, Reverse Transcription, and qRT-PCR Analysis

Transfected cells were harvested for each biological replicate by centrifugation at 200*g* for 5 min and resuspended in 200 μL of RNAlater stabilization reagent (Qiagen) prior to extraction. The total RNA from the transfected cells was extracted using a RNeasy mini kit (Qiagen) according to the supplier’s protocol, and purity was determined by the Nanodrop spectrophotometer 2000 (Thermo Fisher Scientific). The extracted RNA (800 ng) was reverse transcribed using the QuantiTect reverse transcription kit according to the manufacturer’s protocol (including the removal of genomic DNA). The complementary DNA (cDNA) was diluted 10-fold with nuclease-free d.H_2_O to a final volume of 200 μL prior to qRT-PCR analysis. The reaction was set up by mixing 2 μL of diluted cDNA, 2.5 μL of primer mix (combination of forward and reverse primer at a final concentration of 200 nM per primer), 12.5 μL of QuantiFast SYBR green PCR master mix (Qiagen) and 8 μL of nuclease-free d.H_2_O. This was aliquoted in a MicroAmp fast optical 96-well plate (Applied Biosystems, Cheshire, UK). Triplicate reactions were prepared per sample alongside two negative controls, the absence of cDNA template and of reverse transcriptase (for genomic/plasmid contamination). The amplification process was 95 °C for 5 min, followed by 94 °C for 15 s and 60 °C for 60 s over 40 cycles. Melting curve analysis was performed from 60 to 95 °C. The cyclic threshold (Ct) was measured using the 7500 fast real-time PCR system (Applied Biosystems). The mean Ct was calculated per triplicate sample and normalized by comparison against two internal reference gene controls (mmadhc and Fkbp1a).^70^ The primer amplification efficiencies for eGFP, mCherry, and tagBFP mRNA were calculated from standard curves (3-fold and 10-fold serial dilutions). Efficiency was determined by using the equation *E* = 10 (−1/gradient) and was between 93.1 and 97.9% (*r*^2^ > 0.99; Table 1). The cross reactivity of the respective gene primers during amplification were also tested by measuring Ct values when using transient single gene cDNA controls. High Ct values (>29.7) were observed indicating negligible cross reactivity of primers. The absolute quantification of mRNA was performed by interpolating data from gene copy standard curves ranging from 1 × 10^8^ to 6.4 × 10^3^ copies of linearized DNA template for each gene (eGFP, mCherry, and tagBFP).

**Table 1:** Target Gene Primer Sequences and Primer Efficiencies

targeted gene	primer name	primer sequence (5′–3′)	melting point (°C)	GC content (%)	amplicon size (bp)	primer efficiency (%)
eGFP	eGFP-FW-5	ACAAGACCAGAGCCGAAGTG	57.2	55.0	157	96.1
	eGFP-RV-5	TTCTGCTTGTCGGCCATGAT	57.1	50.0		
mCherry	mCherry-FW-2	CCAGTTTATGTACGGCTCCAA	54.8	47.6	106	93.1
	mCherry-RV-2	GTTCATCACTCTCTCCCACTTG	55.1	50.0		
tagBFP	tagBFP-FW-5	CACCTCCTTTCTGTACGGCT	56.8	55.0	305	97.9
	tagBFP-RV-5	CCATGTCGTTTCTGCCTTCC	56.3	55.0		

### Flow Cytometry Analysis

Transfected CHO cells expressing eGFP, mCherry, and tagBFP either as cotransfected SGVs or a MGEV for each biological replicate were harvested after 24 h. The expression was determined by fluorescence using an Attune NxT flow cytometer (Thermo Fisher Scientific). A total of 10 000 viable cells were analyzed per sample. The excitation lasers used to detect fluorescence of tagBFP, eGFP, and mCherry were 405, 488, and 561 nm, respectively, and the emission filters were 440/50, 530/30, and 620/15, respectively. FlowJo software (FlowJo LLC, USA) was employed to apply fluorescent compensation and analyze the data generated. The fluorescent expression was depicted as integrated median fluorescent intensity (iMFI) which is calculated by multiplying the population frequency by the median fluorescent intensity (MFI) for each fluorescent protein.^71^

## Supporting Information Available

*De novo* synthesized vector backbone and transcription units (TUs) for multigene expression vector (MGEV) construction; dynamic range of detection of the coexpression of three fluorescent protein reporters under the control of a low, medium and high strength synthetic promoter at different DNA loads by qRT-PCR; fold change normalized to relative transcriptional activity (RTA) of low strength synthetic promoter expression at each plasmid load (PDF)
