# Supplementary material for: Control of Multigene Expression Stoichiometry in Mammalian Cells Using Synthetic Promoters
Source: ACS Synth Biol. 2021 May 3;10(5):1155–65. doi: 10.1021/acssynbio.0c00643 (PMC8296667; doi:10.1021/acssynbio.0c00643)
Supplement: Supplementary file 1 — sb0c00643_si_001.pdf [file sb0c00643_si_001.pdf]

Control of multigene expression stoichiometry in mammalian cells using synthetic promoters

Yash D. Patel[1,*], Adam J. Brown[1], Jie Zhu[2], Guglielmo Rosignoli[3], Suzanne J. Gibson[4], Diane Hatton[4], David C. James[1,*]

[1]Department of Chemical and Biological Engineering, The University of Sheffield, Mappin St., Sheffield, S1 3JD, UK

[2]Cell Culture and Fermentation Sciences, BioPharmaceuticals Development, R&D, AstraZeneca, Gaithersburg, MD 20878, USA

[3]Dynamic Omics, Antibody Discovery & Protein Engineering, R&D, AstraZeneca, Cambridge, CB21 6GH, UK

[4]Cell Culture and Fermentation Sciences, BioPharmaceuticals Development, R&D, AstraZeneca, Cambridge, CB21 6GH, UK

Supporting Information

The supporting information within this document comprises of three figures namely, Supplementary Figure S1, S2 and S3. Supplementary Figure S1 depicts the vector maps of the *de novo* synthesized vector backbone and transcription units used in the construction of all the multigene expression vectors. Supplementary Figure S2 demonstrates the linear dynamic range of detection of three fluorescent reporter genes when co-expressed under the control of a low, medium and high strength synthetic promoter at different DNA loads by qRT-PCR. Supplementary Figure S3 shows the linear fold change in expression when utilizing a medium and high strength synthetic promoter relative to the low strength synthetic promoter across different transfected DNA loads.

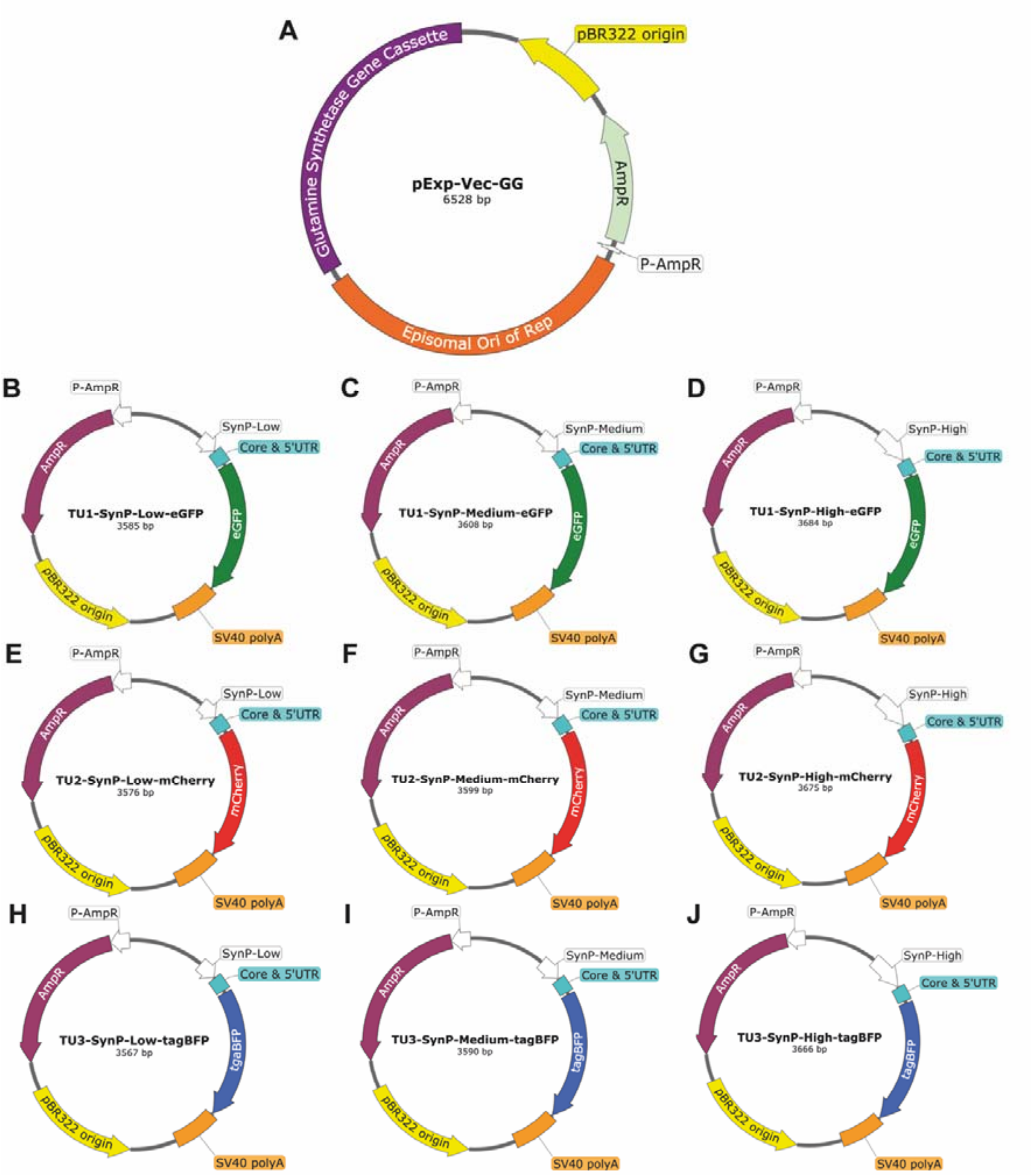

Supplementary Figure S1. *De novo* synthesized vector backbone and transcription units (TUs) for multigene expression vector (MGEV) construction. **(A)** The pExp-Vec-GG vector comprising of a glutamine synthetase (GS) cassette, mammalian episomal origin of replication, β-lactamase gene for ampicillin resistance and microbial origin of replication was used as the backbone to assemble an assortment of TUs to construct a MGEV. **(B-D)** Shuttle plasmids housing the TUs encoding for eGFP under the control of a low **(B)**, medium **(C)** and high **(D)** strength synthetic promoter. **(E-G)** Shuttle plasmids housing the TUs encoding for mCherry under the control of a low **(E)**, medium **(F)** and high **(G)** strength synthetic promoter. **(H-J)** Shuttle plasmids housing the TUs encoding for tagBFP under the control of a low **(H)**, medium **(I)** and high **(J)** strength synthetic promoter. The list of abbreviations used across all the vector maps are as follows: episomal origin of replication (Episomal Ori of Rep), ampicillin resistance gene promoter (P-AmpR), ampicillin resistance gene (AmpR), low strength synthetic promoter (SynP-Low), medium strength synthetic promoter (SynP-Medium), high strength synthetic promoter (SynP-High), core promoter (Core), 5′ untranslated region (5′UTR) and simian virus 40 late polyadenylation element (SV40 polyA). Additionally, the green, red and blue bars represent the eGFP, mCherry and tagBFP coding DNA sequences, respectively. All vector maps were generated using SnapGene® software.

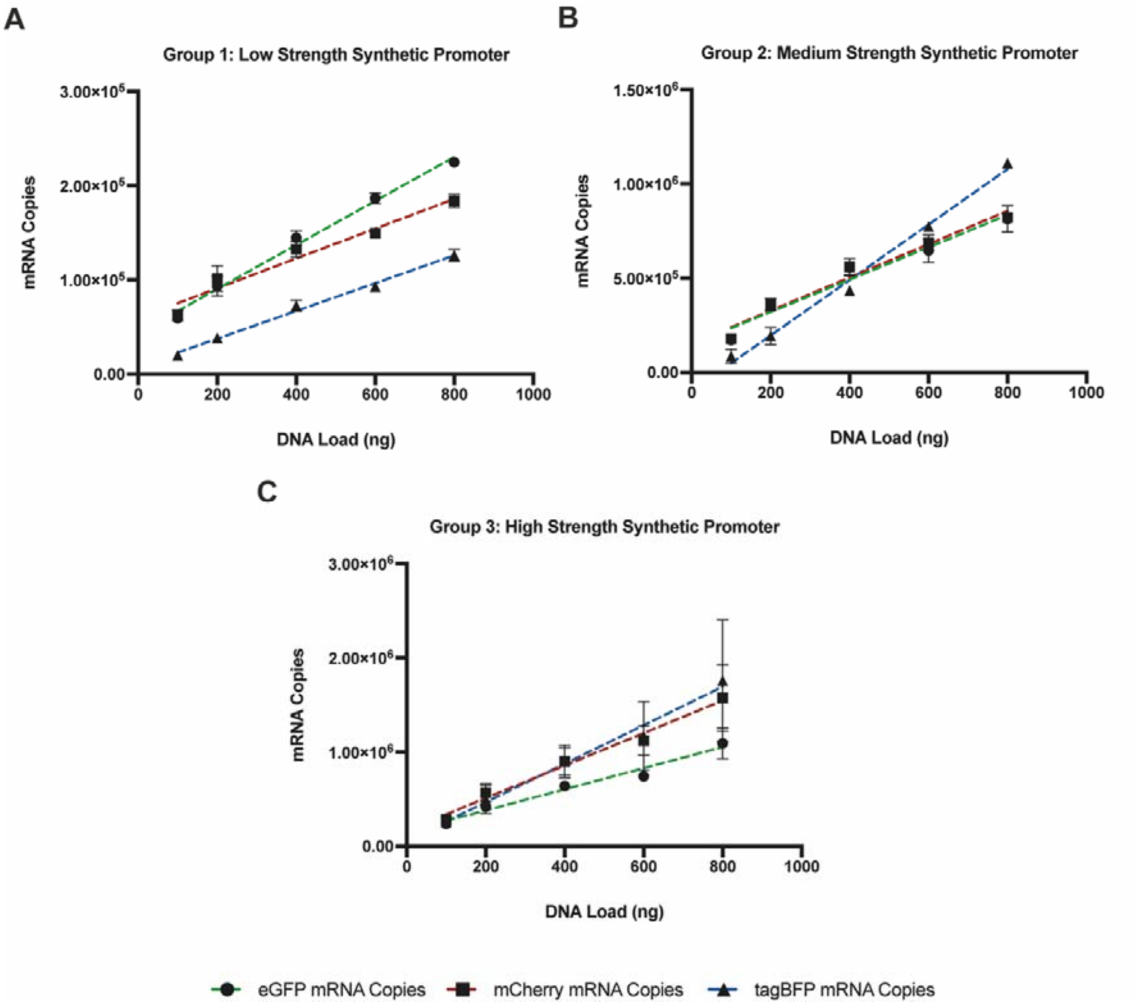

Supplementary Figure S2. Dynamic range of detection of the co-expression of three fluorescent protein reporters under the control of a low, medium and high strength synthetic promoter at different DNA loads by qRT-PCR. **(A-C)** The mRNA copies from the three transfection groups (Group 1: Low strength **(A)**, Group 2: Medium strength **(B)** and Group 3: High strength **(C)**) as described in Figure 1B were quantified at total DNA loads ranging from 100 to 800 ng after 24h. A linear regression curve was fitted between the average mRNA copies and total DNA load for each promoter-reporter combination. The r^2 for all nine curves ranged from 0.953 to 0.992 indicating a linear relationship and stipulating the range of quantification by qRT-PCR. In each plot, the circle, square and triangle points represent average mRNA copies of eGFP, mCherry and tagBFP, respectively and the error bars depict standard deviation from three independent experiments. The green, red and blue dotted lines represent the line of best-fit for eGFP, mCherry and tagBFP, respectively.

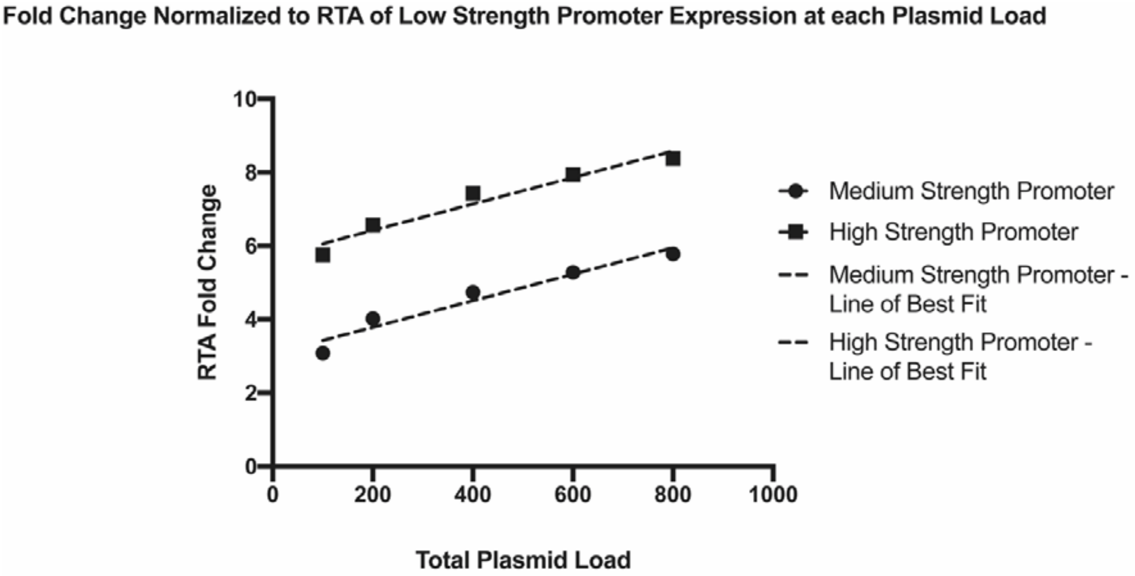

Supplementary Figure S3. Fold change normalized to relative transcriptional activity (RTA) of low strength synthetic promoter expression at each plasmid load. The average fold change in RTA of all three fluorescent protein reporters utilizing the medium and high strength synthetic promoters, relative to the low strength promoter mediated expression, at each transfected plasmid load ranging from 100 to 800 ng. A linear relationship was observed between RTA fold change and total plasmid DNA load transfected when using linear regression. The r^2 of the medium and high strength synthetic promoter mediated expression curves were 0.943 and 0.945, respectively.
